# Long non-coding RNA GAS5 expression in patients with Down syndrome

**DOI:** 10.7150/ijms.45386

**Published:** 2020-05-23

**Authors:** Michele Salemi, Giovanna Marchese, Angela Cordella, Rossella Cannarella, Concetta Barone, Maria Grazia Salluzzo, Aldo E. Calogero, Corrado Romano

**Affiliations:** 1 Oasi Research Institute-IRCCS, Troina (EN), Italy.; 2 Genomix4Life Srl, Department of Medicine, Surgery and Dentistry “Scuola Medica Salernitana”, University of Salerno, Baronissi (SA), Italy.; 3 Department of Clinical and Experimental Medicine, University of Catania.95123 Catania. Italy.

**Keywords:** Down Syndrome, lncRNA *GAS5*, expression, qRT-PCR, RNA sequencing

## Abstract

Trisomy 21, also known as Down Syndrome (DS), is the most common chromosome abnormality and causes intellectual disability. Long non-coding RNA (lncRNA) growth arrest-specific 5 (*GAS5*), whose differential expression has recently been reported in patients with Klinefelter syndrome, has been addressed to play a role in the development of inflammatory and autoimmune diseases, vascular endothelial cells apoptosis and atherosclerosis, all being common features in patients with DS. Therefore, the aim of this study was to assess the lncRNA *GAS5* expression profile in DS patients and in controls. lncRNA *GAS5* levels were evaluated by qRT-PCR assay in 23 patients with DS and 23 age-matched controls. A significant lncRNA *GAS5* down-regulation was observed in patients with DS by RT-PCR analysis, The RNA sequencing experiments confirmed the qRT-PCR data. LncRNA *GAS5* down-expression may play a role in the development of some typical features of the patients with DS and, particularly, in inflammatory and autoimmune diseases.

## Introduction

Down Syndrome (DS), caused by a trisomy of chromosome 21, is a common genetic disorder (1:600-700 newborns). The simple trisomy is due to an abnormal chromosome separation at the first or second meiotic division leading to a doubled chromosome 21 in the reproductive cells (in the oocytes for the 95% of cases). The transfer of a chromosome fragment to another (usually between chromosomes 14 and 21, 21 and 22) results in translocation trisomy and occurs in about 5-6% of patients with DS 1. The trisomy leads to a developmental disorder causing intellectual disability, early onset of Alzheimer's disease, cardiovascular defects, nutritional diseases (overweight, obesity, hypercholesterolemia and deficiencies of vitamins and minerals)^2^, dysfunction of the muscle-skeletal and gastrointestinal systems, abnormal immune function, endocrine disruption of the hypothalamic-pituitary-thyroid axis, and the appearance of various phenotypic features such as upslanted palpebral fissures, flat nose and short stature^3^ . The expression of chromosome 21 genes is dynamic and complex 4. Furthermore, genes mapping on the other chromosomes have been suggested to play a role in DS phenotype 5. The molecular mechanisms underlying the variability in DS phenotype have not been clearly understood yet. Growth arrest-specific 5 (*GAS5*) gene, mapping on 1q25.1 chromosomal band, encodes for a long non-coding RNA (lncRNA) that is involved in the modulation of gene expression targeting many different downstream miRNAs. lncRNA* GAS5* was initially identified as a tumor suppressor gene 6. Recently, it has been shown to be over expressed in patients with Klinefelter syndrome^7^. Furthermore, recent evidence suggests that lncRNA GAS5 plays a role in autoimmune disorders 8, widely recognized as important DS comorbidities. To the best of our knowledge, no study evaluated the lncRNA GAS5 expression profile in patients with DS. Therefore, the aim of this study was to assess lncRNA GAS5 expression in fibroblasts from DS subjects, compared to those of fibroblasts from Normal Controls (NC) subjects by qRT-PCR. Furthermore, preliminary data on a study of the transcriptome in peripheral blood mononuclear cells (PBMCs) of subjects with DS have been included.

## Materials and Methods

### Samples for qRT-PCR and RNA sequencing

A total of 46 subjects, including 23 DS patients (12 males and 11 females; age range 25-57 years) and 23 NC (12 males and 11 females; age range 22-55 years) for qRT-PCR experiments (Table 1), were recruited at the Oasi Research Institute - IRCCS (Troina, Italy). Human gingival fibroblasts were isolated from explants of human gingiva and cultured in Dulbecco's modified Eagle's medium as described by Salemi et al. (2015) 9, three are the passages to which the fibroblasts are subjected. Blood samples were with drawn from each patient and control (Table 2) for Next Generation Sequencing (NGS) analysis, were recruited at the Oasi Research Institute - IRCCS (Troina, Italy). The study was approved on 3 June 2017 by the local IRB (2017/05/31/CE-IRCCS-OASI/9). All the study participants, or their legal guardians, signed an informed consent to publish.

### Total RNA extraction for qRT-PCR

RNA extraction by human gingival fibroblasts and retro-transcription were performed as previously described using RNeasy mini Kit (Qiagen Sciences, Germantown, USA) 9. RNA was quantified using Nanodrop ND-1000 spectrophotometer (NanoDrop Technologies). The RNA samples were stored at -80°C till further use. Genomic DNA elimination reaction was performed using QuantiTect Reverse Transcription Kit (Qiagen, Germany), with thermocycler program: 2' min at 42°C. Reverse-transcription (cDNA synthesis) was carried out using 100ng of RNA and QuantiTect Reverse Transcription Kit (Qiagen Sciences, Germantown, USA), thermocycler program: 15' at 42°C and 3' at 95°C.

### Total RNA extraction for RNA sequencing

PBMCs were separated from each blood sample using Ficoll-Paque (Ficoll Plaque PLUS - GE Healthcare Life Sciences, Piscataway, USA) and the RNA was extracted using TRIzol reagent (TRIzol Reagent, Invitrogen Life Tecnologies, Carlsband, CA, USA) according to the manufacturer's instructions. The RNA obtained was stored at -80ºC.

### Real-time quantitative PCR (qRT-PCR)

We compared qRT-PCR in 23 DS patients and 23 normal subjects. QRT-PCR experiments were performed using the Light Cycler 480 (Roche Diagnostics; Mannheim, Germany) in a total volume of 25 μl. The lncRNA GAS5 target gene assay (assay ID Hs03464472_m1) and the glyceraldehyde-3-phosphate dehydrogenase (GAPDH) reference gene assay (assay ID Hs99999905_m1) were obtained from Applied Biosystems (Carlsbad, CA, USA). The thermal cycling conditions consisted of one cycle of 2 min at 50°C (UDG incubation), one cycle of 15 min at 95°C (enzyme activation) and 42 cycles of 15 seconds at 94°C followed by 1 min at 60°C (PCR). The kit used was QuantiTect probe PCR Kit (Qiagen Sciences, Germantown, USA). The amplified transcripts were quantified using the threshold cycle (Ct) method and relative quantification analysis data were played using comparative ΔΔCt method: each cDNAfrom DS subjects was coupled with cDNA from normal subjects with the same sex and age, more or less three years. Light Cycler 1.5 software supplied with Light Cycler 480 was used for relative quantification analysis.

### Statistical analysis for qRT-PCR

Distribution analysis of lncRNA GAS5 levels was performed using Shapiro - Wilk test and inferential statistical analysis was carried out using Wilcoxon rank-sum test and bivariate linear regression analysis. Graph Pad Prism 5 software was used for statistical analysis. A *p* value lower than 0.05 was considered significant.

### RNA sequencing and data analysis

RNA-Sequencing was performed by Genomix4Life srl (Baronissi (SA), Italy). RNA concentration in each sample was assayed with a ND-1000 spectrophotometer (NanoDrop) and its quality assessed with the TapeStation 4200 (Agilent Technologies). Indexed libraries were prepared from 1 µg/ea purified RNA with TruSeq Stranded Total RNA (Illumina) Library Prep Kit according to the manufacturer's instructions. Libraries were quantified using the Agilent 2100 Bioanalyzer (Agilent Technologies) and pooled such that each index-tagged sample was present in equimolar amounts, with final concentration of the pooled samples of 2 nM. The pooled samples were subject to cluster generation and sequencing using an Illumina NextSeq 500 System (Illumina) in a 2x75 paired-end format. The raw sequence files generated (.fastq files) underwent quality control analysis using FastQC (https://www.bioinformatics.babraham.ac.uk/projects/fastqc/). Bioinformatics analysis were performed by Genomix4Life Srl (Baronissi, SA, Italy). The quality checked reads were trimmed with cutadapt v.1.10 and then aligned to the human genome (hg38 assembly) using STAR v.2.5.2 10, with standard parameters. Differentially expressed mRNAs were identified using DESeq2 v.1.12 11. The sequencing data are available upon request. As partial data of a larger research project not yet completed.

## Results

**Figure 1:**
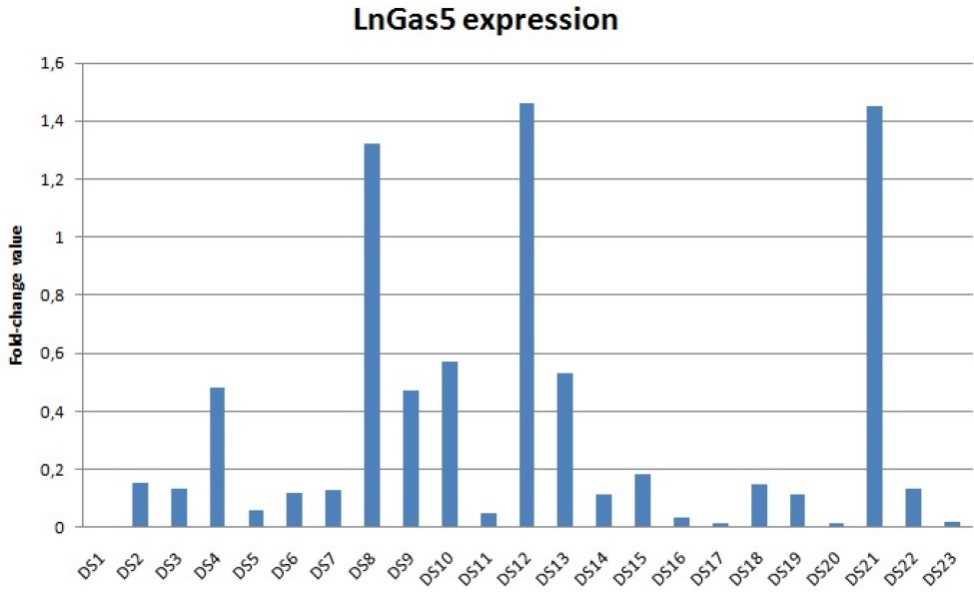
lncRNA GAS5 expression in subjects with Down syndrome compared to normal controls (the value for controls is always 1 - not included in the table) . Data shown were obtained by qRT-PCR and comparative ΔΔCt method; DS: Down syndrome; NC: normal controls.

**Figure 2:**
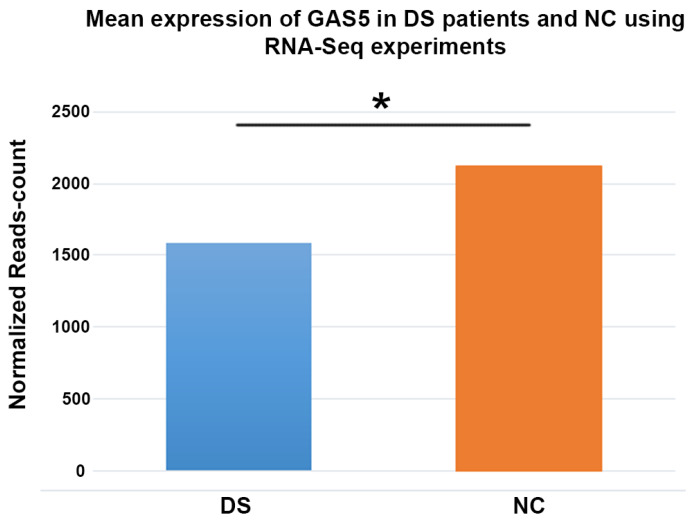
Histogram showing the mean expression of lncRNA GAS5 from RNA sequencing experiments in subjects with Down syndrome compared to normal controls. DS: Down syndrome; NC: normal controls. * denotates a *p-value* < 0,001

We found a decreased lncRNA GAS5 levels in 22 out of 23 patients with DS; 15 DS showed values lower than 10% compared to the relative control (Figure 1). The distribution of the expression values was found to be not normal (*p*<0.01) and inferential statistical analysis revealed a significant difference between the two groups: DS vs. controls (*p*<0.001). No significant statistical difference was found in two groups according to gender and age (*p*>0.05). RNA sequencing (RNA-Seq) data obtained from 6 DS patients and 5 NC showed how lncRNA GAS5 is down-regulated in the patients with DS respect to the controls (Figure 2).

## Discussion

LncRNA GAS5 has been reported to play a role in inflammatory and autoimmune disorders, apoptosis and glucocorticoid (GC) actions 8,12. In fact, one of the mechanisms by which lncRNA GAS5 could be involved in the development of autoimmune disorders may be its influence on the glucocorticoid receptor (GCR) function. Accordingly, this non coding RNA is capable of direct interaction with the DNA binding domain of the GCR 13. Altered lncRNA GAS5 expression in patients with rheumatoid arthritis, systemic lupus erythematosus, osteoarthritis and inflammatory bowel disease has been described, both up-regulation and down-regulation mechanisms have been highlighted, these mechanisms directly affect the GCR that are involved in this type of pathology 8. All this is consistent with the possible involvement of lncRNA GAS5 in the pathogenesis of inflammatory and autoimmune diseases, more frequently occurring in patients with DS.

Patients with DS are at risk of metabolic disorders and endothelial dysfunction 14. LncRNA GAS5 over-expression triggers vascular endothelial cells apoptosis after lipoperoxidation. Both human and animal models show higher levels of lncRNA GAS5 in plaques of atherosclerosis compared to healthy vessels. Therefore, its expression may likely be involved in atherogenesis and may represent an interesting target for the treatment of atherosclerosis 15. Other important experimental data is that LncRNA GAS5 expression seems to be involved in vasculopathy-related processes, thus representing a target for atherosclerosis-related treatments 16. The role of lncRNA GAS5 in atherogenesis in DS patients deserves elucidation but we cannot exclude that lncRNA GAS5 dysregulation may play a role, furthermore it could also be assumed that the same mechanism may affect early dementia in DS patients. Finally, ovarian reserve is reduced in patients with DS 14. LncRNA GAS5 seems to be involved the induction of cell apoptosis 12,17 and, therefore, might trigger ovarian cell apoptosis. The possible role of lncRNA GAS5 over-expression in the pathogenesis of reduced ovarian reserve in patients with DS may need to be further explored.

Various studies have shown that lncRNA GAS5 also plays a role in the brain, in fact lncRNA GAS5 can effectively inhibit the proliferation, migration and invasion of glioma cells and promote cell apoptosis through targeting GSTM3 expression 18. Moreover a study highlights that silencing GAS5 may inhibit neuronal apoptosis and improve neurological function in ischemic stroke, which contributes to better understanding of the pathologies of ischemic stroke and development of novel therapeutic options for this disease 19. If lncRNA GAS5 was down-expressed in the brain of DS subjects during embryonic development, it could influence normal brain development, for example by acting on the apoptotic mechanisms that are necessary for the same embryonic development.

In conclusion, we found that lncRNA GAS5 is down regulated in patients with DS compared to NS. Given that lncRNA seems to be involved in various biologic processes, such as GC actions, inflammatory and autoimmune diseases, apoptosis and atherosclerosis, we speculate that lncRNA GAS5 decreased expression may be involved in the some typical phenotypes of DS patients.

## Figures and Tables

**Table 1:** Table with data on patients and controls involved in qRT-PCR experiments.

Subjects DS	Sex	Age	Subjects NC	Sex	Age
DS1	F	29	NC1	F	30
DS2	F	38	NC2	F	37
DS3	F	34	NC3	F	31
DS4	F	27	NC4	F	30
DS5	M	23	NC5	M	22
DS6	F	27	NC6	F	24
DS7	M	34	NC7	M	32
DS8	F	23	NC8	F	25
DS9	M	24	NC9	M	24
DS10	M	26	NC10	M	25
DS11	M	25	NC11	M	24
DS12	F	25	NC12	F	22
DS13	M	30	NC13	M	33
DS14	M	57	NC14	M	55
DS15	M	35	NC15	M	33
DS16	F	27	NC16	F	25
DS17	M	29	NC17	M	30
DS18	F	42	NC18	F	44
DS19	M	41	NC19	M	38
DS20	M	31	NC20	M	29
DS21	F	40	NC21	F	37
DS22	M	36	NC22	M	37
DS23	F	25	NC23	F	26

DS: Down syndrome; NC: normal controls.

**Table 2:** Table with data on patients and controls involved in RNA sequencing experiments.

Subjects DS	Sex	Age	Subjects NC	Sex	Age
1 DS	F	29	1 NC	F	30
2 DS	M	26	2 NC	M	25
3 DS	M	25	3 NC	M	24
4 DS	F	25	4 NC	F	22
5 DS	M	29	5 NC	M	30
6 DS	F	42			

DS: Down syndrome; NC: normal controls.
